# Effect of the *FoodSwitch* application on type 2 diabetes in Sweden: a study protocol for the randomised controlled DIgitAl diabeTES Treatment – the Healthy Eating, heaLthy Patients trial (DIATEST-HELP)

**DOI:** 10.1136/bmjopen-2025-110141

**Published:** 2025-11-16

**Authors:** Peder af Geijerstam, Emir Johansson, Siri Fägerstam, Jason HY Wu, Bijar Ghafouri, Karin Karlsson, Lana Hebib, Lisa Kastbom, Patrik Wennberg, Emelie Gustafson Hedov, Maria Storgärds, Johan Sundström, Karin Rådholm

**Affiliations:** 1 Department of Health, Medicine, and Caring Sciences, Linköping University, Linköping, Sweden; 2 Primary Healthcare Center Cityhälsan Centrum, Region Östergötland, Norrköping, Sweden; 3 Primary Healthcare Center Kärna, Region Östergötland, Linköping, Sweden; 4 The George Institute for Global Health, University of New South Wales, Sydney, New South Wales, Australia; 5 Department of Medical Sciences, Uppsala University, Uppsala, Sweden; 6 Primary Healthcare Center Sandbyhov, Region Östergötland, Norrköping, Sweden; 7 Primary Healthcare Center Ekholmen, Region Östergötland, Linköping, Sweden; 8 Department of Public Health and Clinical Medicine, Umeå University, Umeå, Sweden; 9 MinForskning AB, Uppsala, Sweden

**Keywords:** Diabetes Mellitus, Type 2, NUTRITION & DIETETICS, Mobile Applications, Randomized Controlled Trial

## Abstract

**Introduction:**

A healthy diet improves glycaemic control and reduces cardiovascular risk in type 2 diabetes (T2D). However, access to dietitians is limited. Several countries have implemented mandatory interpretive front-of-pack labelling to guide consumers towards healthier food choices, but Sweden has not. Smartphone applications may offer an alternative platform to provide such information. This study evaluates the dietary and clinical impact of a novel application providing interpretive labelling to Swedish adults with T2D.

**Methods and analysis:**

This is a fully decentralised randomised controlled trial. 900 individuals with T2D for ≥2 years who regularly shop for groceries will be recruited via general practices and community advertisements. Participants will be randomised to receive either: (1) access to the *FoodSwitch* mobile application plus standard written dietary advice, or (2) standard written dietary advice only. The *FoodSwitch* application allows users to scan barcodes on packaged foods to receive recommendations of healthier alternatives within the same category. The primary outcome is the difference in change in mean self-measured glycated haemoglobin between groups after 6 months. Secondary outcomes include differences in changes in waist circumference, body weight, quality of life, medication use, hospitalisations and all-cause mortality at 26 weeks. Exploratory outcomes include omics analyses. Recruitment is ongoing. Expected study completion on 31 December 2026.

**Ethics and dissemination:**

The trial has received ethical approval from the Swedish Ethical Review Authority (2023-06622-01, 2024-06668-02, 2024-07357-02 and 2025-01095-02) and is performed in line with World Medical Association Declaration of Helsinki and the General Data Protection Regulation. Results will be published in a peer-reviewed international journal.

**Trial registration number:**

NCT05977218.

**STRENGTHS AND LIMITATIONS OF THIS STUDY**

- The study is fully decentralised, enabling national recruitment and participation without site visits.
- Use of digital consent, self-administered anthropometrics and dried blood spot sampling minimises participant burden and costs.
- The intervention is based on an evidence-informed, widely available mobile platform (*FoodSwitch*) adapted to the Swedish food market.
- Limitations include self-reported dietary and anthropometric data, potential selection bias towards digitally literate participants and inability to blind participants to allocation.

## Introduction

### Background and rationale

Diabetes, a major risk factor for cardiovascular disease, affects approximately 6.2% of the Sweden population, with 88% of cases managed in primary care.^1 2^ Obesity and lifestyle factors are key drivers in the development of type 2 diabetes (T2D), and with over half of adults in Sweden overweight or obese, the prevalence continues to rise.^3 4^ Two out of five persons with T2D in Sweden are overweight, and another two out of five are obese.^2^ Dietary change is a first-line recommendation in clinical guidelines as it can reduce body weight, improve glycaemic control and lead to T2D remission.^3^ However, many individuals struggle to follow dietary advice.^5^

The WHO recommends mandatory front-of-pack nutrition labelling (FOPL) as a key public health strategy to promote healthier diets and reduce the burden of non-communicable diseases, which are the leading causes of death globally.^6^ Yet in Sweden, no mandatory FOPL system has been implemented. Furthermore, access to dietitians is limited.^7 8^ The voluntary Keyhole label is displayed exclusively on the healthiest products in each food category, thereby limiting its potential to influence consumer choices across the broader product spectrum. Selecting healthy products can be challenging due to the wide range of available products, and real-world interventions suggest that interpretive labelling improves dietary choices.^9^

Smartphone-based interventions for health behaviour change and disease management offer key advantages, including low cost and broad scalability,^10 11^ and 99% of Swedes own a mobile phone.^12^ Studies using smartphone applications, text messaging or telehealth video conferencing to support healthy eating have shown promising results for weight loss in overweight and obese adults.^13^ Numerous applications aimed at promoting healthy eating, cooking and weight control are available, but the majority have not been evaluated for their effectiveness.^14^ While mobile health (mHealth) tools have been shown to improve diet and clinical management in patients with T2D,^15^ no evidence-based such tools for dietary advice are currently available in Sweden. Sweden’s high digitalisation (99% smartphone ownership) and lack of mandatory FOPL make it an ideal setting for testing scalable digital dietary interventions.

### Objectives

The aim of the current study is to evaluate the effect of a novel smartphone app (*FoodSwitch),* in addition to standard written dietary advice, compared with standard written dietary advice alone, on blood glucose control (glycated haemoglobin, HbA1c) in individuals with T2D.

## Methods and analysis

### Study design and timeline

This will be a trial of 900 participants randomised to *FoodSwitch* or control (1:1 allocation) with a 26-week follow-up (table 1). The study will be fully decentralised, with recruitment, inclusion and data collection all performed remotely. Recruitment started on 25 August 2025 and is estimated to continue until 31 December 2026. Study completion is estimated to be 31 December 2031.

**Table 1:** Schedule of enrolment, interventions and assessments

	Screening	Run-in period 1	Run-in period 2	Postallocation period				Long-term follow-up
	0–4 weeks prior	0–3 weeks prior	0–6 weeks prior	0	6 weeks	13 weeks	26 weeks	3–7 years
Enrolment								
Digital consent	X							
Screening questionnaire	X							
Randomisation				X				
Intervention and control								
The *FoodSwitch* app				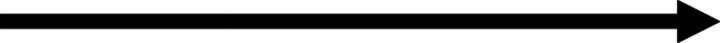				
Standard written dietary advice				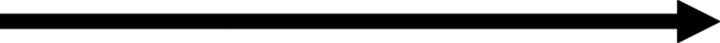				
Assessments								
Questionnaires		X			X	X	X	
Collection of receipts		X				X	X	
Anthropometric measurements			X			X	X	
Blood samples			X			X*	X	
National register data							X	X

*Week 13, only glycated haemoglobin.

### Inclusion and exclusion criteria

Inclusion criteria will be age between 18 and 75 years, self-reported T2D with a duration of ≥2 years and does grocery shopping at physical grocery stores. Exclusion criteria will be no access to *BankID* (a secure electronic identification system, widely used in Sweden) or failure to complete eligibility screening and run-in periods 1 (questionnaires and grocery receipt collection) and 2 (self-measurement of waist circumference, body weight and self-collection of capillary blood samples).

### Recruitment

Participants will be recruited by clinicians in primary care, as well as through social media, patient associations, letters and newspaper advertisements. Individuals from all parts of Sweden will be invited to participate.

Individuals interested in participating will be directed to *minforskning.se* (MinForskning AB, Uppsala, Sweden), an online platform for digital consent.^16^ The platform will present detailed written information about the study, including the study’s purpose, risks and benefits and participants’ right to withdraw at any time, and allow individuals to provide digital consent (online supplemental material). The platform uses *BankID* (Finansiell ID-Teknik BID AB, Stockholm, Sweden), a secure digital identification system accessible to 99.3% of Swedish adults 75 years or younger (Personal Communication with BankID Customer Support, 12 August 2025). No study-specific procedures will be performed before consent has been provided. *Minforskning.se* will securely store the signed consent forms and provide participants with copies on request. Participants can withdraw their consent to participate at any time, with no influence on further clinical care or treatment. Data collected before any withdrawal are retained and included in analyses.

10.1136/bmjopen-2025-110141.supp1 Supplementary data

10.1136/bmjopen-2025-110141.supp2 Supplementary data

After providing consent, participants will be asked to complete a questionnaire to confirm eligibility. Those who respond within 28 days may proceed to run-in period 1.

### Screening eligibility and run-in periods

In run-in period 1, participants will complete questionnaires on demographics, household characteristics, grocery purchasing habits, medical history and medications and upload grocery shopping receipts from 1 week of typical grocery purchases. Reminders to complete the questionnaires and upload receipts will be sent after 7 and 14 days. Participants who complete these tasks within 21 days may proceed to run-in period 2.

In run-in period 2, participants will be asked to measure their waist circumference and body weight and to self-collect capillary blood on filter papers (see the Assessments section). Participants who complete the capillary blood test within 42 days will proceed to randomisation.

### Randomisation

Participants will be randomised to the intervention or control in a 1:1 ratio using REDCap (Vanderbilt University, Nashville, Tennessee, USA) based on a computer-generated randomisation list. Healthcare providers, study investigators and data analysts will be blinded, while participants will not be blinded.

### Intervention

**Figure 1:**
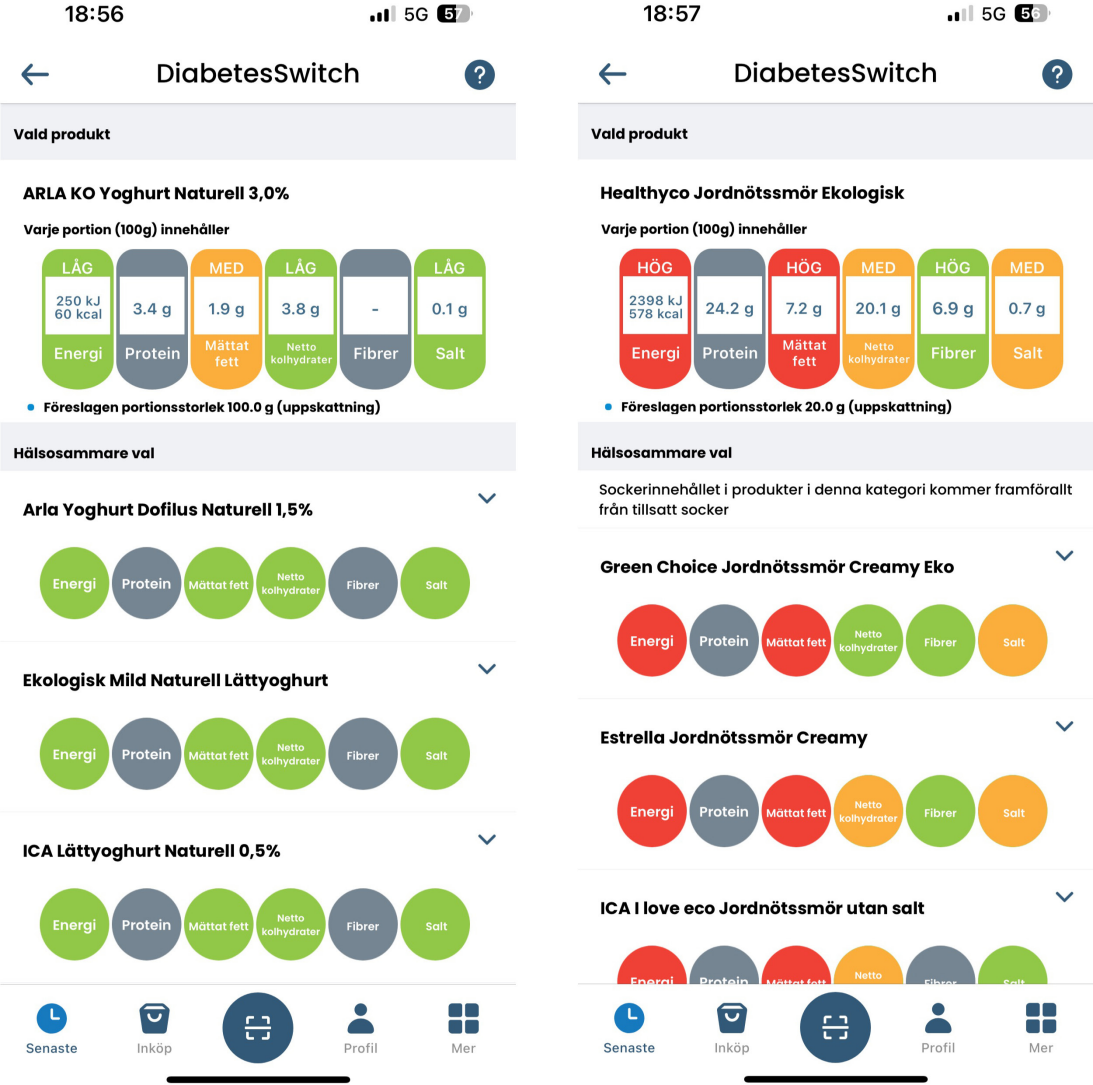
Two screenshots from the *DiabetesSwitch* mobile application. The app evaluates a selected food product (top section) based on five nutritional components: energy, protein, saturated fat, net carbohydrates, fibre and salt, using a traffic-light colour-coded rating system (green=better, yellow=intermediate, red=worse and grey=not available or not rated). Below the selected product, the app suggests alternative options with more favourable nutritional profiles, using the same visual scale to allow for direct comparison across all components.

Participants randomised to the intervention group will receive access to the *FoodSwitch* app with the *DiabetesSwitch* filter for 26 weeks, in addition to standard dietary advice (see the Control section). The *FoodSwitch* platform, developed by The George Institute for Global Health (Sydney, Australia), includes a database of over 700 000 products across 12 countries. When grocery shopping, the app is used to scan barcodes on packaged food items, providing interpretive nutritional information using a traffic light colour-coded ranking based on energy, fat, sugars and salt content and suggesting alternative products, within the same product category, with better nutritional quality (figure 1). For this trial, data from the Dabas open database^17^ (Bayn Solutions AB, Stockholm, Sweden) and the three largest grocery store retailers in Sweden, representing 70.9% of the market and collected through website scraping, were processed to incorporate the nutrient profiles of approximately 57 000 Swedish food products into the platform database.^18^ Food products were then manually categorised at the product level to enable the identification of healthier alternatives within the same category. In cases where categorisation was unclear, two research assistants independently assessed the product to determine the most appropriate classification.

The *DiabetesSwitch* filter, also developed by The George Institute for Global Health, is adapted for individuals with T2D and integrated into the *FoodSwitch* app. *DiabetesSwitch* tailors nutrient displays and recommendations for individuals with T2D. While *FoodSwitch* displays energy, saturated fat and salt, *DiabetesSwitch* also includes proteins, net carbohydrates and fibre, disqualifying products with low fibre content, thereby supporting better glycaemic control. The app functions by scanning a product’s barcode, identifying its nutrient profile from a database of manufacturer-provided on-pack data, and ranking similar products based on the sum of total energy and energy from net carbohydrates, with lower totals ranked as healthier.

#### Control

Participants randomised to the control group will receive standard written dietary advice in the form of a pamphlet widely used in Swedish primary care, sent via postal mail.^19^

### Endpoints

#### Primary endpoint

Difference in change in mean HbA1c between intervention and control at 26 weeks will be examined using linear regression. Models will be adjusted for baseline characteristics, including age, sex and baseline values, to increase power, as advised by regulatory authorities.^20^

#### Secondary endpoints

Difference in change in national registry-based data on medication use, hospitalisations, outpatient visits and diabetes complications, self-measured waist circumference and body weight, self-reported quality of life and dietary self-efficacy, between intervention and control at 26 weeks, will be examined using linear regression. Difference in all-cause mortality between intervention and control at 26 weeks will be analysed using Cox proportional hazards models. Registry-based outcomes will also be assessed and compared between intervention and control at 2 and 5 years, respectively. Additionally, changes in patterns of food purchasing, identified from receipts and focusing on fibre-rich foods (such as legumes, vegetables, fruits, nuts and whole grains), processed foods, dairy products (low- and full-fat) and confectionery, between intervention and control at 13 and 26 weeks, will be examined using linear regression.

#### Exploratory endpoints

The omics data include proteomics and metabolomics, which may reveal biological mechanisms underlying the dietary intervention. These data will be analysed using advanced, multivariate data analysis,^21^ which has the potential to identify a pool of biomarkers with superior diagnostic and prognostic performances compared with a single-marker approach. These exploratory analyses will be specified after the conclusion of the study and may include partial least squares regression analyses, cluster analysis and principal component analysis. Before such analysis, principal component analysis will be used to check for multivariate outliers. The guidelines for interpreting and presenting results presented by Wheelock and Wheelock will be followed.^21^ Moreover, network construction to organise and make sense of the large amount of omics data will be performed using bioinformatics in order to find functions, pathways or networks involved in the biological mechanisms affected by the intervention.

### Assessments

Data collection will be performed in a decentralised manner, through the use of digital tools and blood samples sent by post, coordinated at Linköping University, Sweden. All participants will be asked to provide the following information.

#### Questionnaires

Participants will be asked to fill in questionnaires via *Symptoms* (Symptoms Europe AB, Uppsala, Sweden), a validated health data collection online platform accessible via smartphones, tablets and computers.^22^ Patient-reported outcome measures have been used sparingly in clinical practice, but digital versions are as effective, or more effective than paper forms.^23 24^ Advantages include simplified data management, improved completion rates through digital reminders and novel data types, such as three-dimensional pain drawings.^24–26^

Questionnaires cover quality of life (EQ-5D-5L), digital tool usage, grocery shopping practices and self-efficacy, sleep patterns, physical activity, dietary habits, alcohol and tobacco use, as well as open-ended symptom reporting and participant-generated symptom drawings. These questionnaires will be collected during run-in period 1 and at 13 and 26 weeks during the postallocation period, and EQ-5D-5L will also be collected at 6 weeks during the postallocation period. During run-in period 1, a medical history questionnaire will also be collected. Some questionnaires will also be available for spontaneous completion at any time as chosen by the participant.

#### Anthropometrics

Via postal mail, participants will receive a tape measure with written instructions for self-measurement of waist circumference. Those with access to a home scale will also receive instructions for measuring body weight. For participants with missing self-reported weights, data from the Swedish National Diabetes Register will be used. Measurements will be collected during run-in period 2 and at 13 and 26 weeks during the postallocation period.

#### Blood samples

Participants will receive a blood sample collection kit from Vitas Analytical Services AS (Oslo, Norway) containing an alcohol swab, two lancets, two filter papers (each with four collection spots) and a return envelope. The kit will be sent during run-in period 2 and at 13 and 26 weeks during the postallocation period. After an 8-hour overnight fast, participants will be instructed to collect capillary blood droplets onto each designated filter paper spot via finger prick and return the dried samples by postal mail.

For the collection of the dried blood spots on filter papers, the participants will thoroughly wash and disinfect their hands with alcohol, followed by air drying. A lancet will then be used to puncture the third or fourth digit, and the initial drop of blood formed will be discarded. The finger will then be gently massaged to facilitate the formation of a sufficient blood droplet, which will be dropped onto the first circle of the filter paper, ensuring no contact with the surface of the filter paper. Subsequently, the finger will again be massaged to produce additional droplets, which will be used to fill the remaining circles of the filter papers. Following sample collection, the blood cards will be left to air dry at ambient room temperatures for 3 hours, after which they will be stored at −80°C until further analysis.

A novel collection of capillary blood onto filter paper using a lancet offers advantages to venepuncture, including minimal invasiveness, low cost, no need for healthcare personnel and simplified transport via regular mail.^27 28^ Dried blood spots on filter paper have been used in several large-scale studies, and HbA1c can be reliably estimated.^28–33^ In the 12-week DIATEST-HELP (DIgitAl diabeTES Treatment – the Healthy Eating, heaLthy Patients) pilot study, almost 90% of participants were comfortable with performing the self-administered blood test.^34^

### Register data

Several national Swedish registers will be used for long-term follow-up of cardiovascular outcomes and diabetes complications, as well as detailed baseline characteristics. The Swedish National Patient Register will be used to collect data on hospitalisations and hospital outpatient visits, including patient demographics, caregiver information, date of admission/discharge or visit, diagnosis (primary and secondary) and clinical procedures; The Swedish Cause of Death Register will be used to collect data on dates and underlying causes for all deaths; The Swedish Prescribed Drug Register will be used for information on all dispensed drug units including drug identification number, number of dispensed units, costs, patient demographics (including age and sex), date of prescription and dispensation and code for caregiver; The Swedish National Diabetes Register will be used for data on individuals with diabetes, including treatment, risk factors (eg, blood lipids, physical activity, office blood pressure) and relevant complications (eg, diabetic foot ulcers, retinopathy, kidney function).

### Sample size

A total of 714 participants (357 in each arm) will provide 80% power (two-sided, α=0.05) to detect a 3 mmol/mol difference in change in HbA1c between intervention and control groups, assuming an SD of 14 mmol/mol.^35^ A 3 mmol/mol difference was selected as a clinically meaningful effect, comparable to that achieved with commonly prescribed glucose-lowering medications such as thiazolidinediones, biguanides and sodium-glucose cotransport-2 inhibitors.^36^ To allow for up to 20% drop-out, 900 participants (450 per arm) will be recruited.

### Data management

During the study, data will be collected in the respective systems. Sent and received test kits, as well as data from *Symptoms*, will be recorded in REDCap. After the completion of the study, these data will be merged into a single database. If applicable, missing data will be addressed using listwise deletion or imputation, depending on the degree and pattern of missingness.

Participant inclusion and data collection will be overseen by the study investigators as needed, independently of the sponsor and funders. A formal data monitoring committee will not be established, as the study does not involve the evaluation of a pharmaceutical intervention.

### Blood samples

Filter papers with dried blood spots will be stored at −80°C at the biobank facility at Region Östergötland (Linköping, Sweden).

Analyses of HbA1c will be performed by Vitas AS (Oslo, Norway). The results of the HbA1c analyses will be reported back to the study participant on the web-based clinical trial platform *minforskning.se* at the end of the trial. Dried blood spot sampling for HbA1c has been validated against venous blood with high correlation and narrow 95% limits of agreement.^37^

DIATEST-*Dietomics* analyses of proteomics and metabolomics (including fatty acids) will be performed at the Science for Life Laboratory and PAINOMICS laboratory at Linköping University (Linköping, Sweden). Different extraction methods using various buffer solutions will be tested to optimise the extraction of proteins and metabolites from the dried blood spots. Proteomic analyses will be performed using two-dimensional gel electrophoresis or liquid chromatography in combination with tandem mass spectrometry. Metabolomic analyses will be performed using nuclear magnetic resonance spectroscopy. Targeted inflammatory and metabolic protein analysis such as cytokines/chemokines and glucagon, insulin and leptin will be performed using multiplex immunoassay analysis using meso scale discovery electrochemiluminescence technology.

Molecular biomarkers include gene, protein, metabolite and lipid biomarkers. Omics methods include large scale data analysis to characterise and quantify the total pools of biological molecules. Such methods have been widely used for identification of potential biomarkers in several disease conditions such as neurological disorders, heart diseases and cancer.^38^ Proteomics uses mass spectrometry to assess protein structure, function and interactions, offering insights beyond genomics. With nearly a million proteins, it is complex but valuable for precision medicine.^39^ Metabolomics is the comprehensive analysis of all metabolites in a cell and can be used to evaluate dietary patterns.^40 41^ Since a large number of substances can be analysed simultaneously, omics is potentially a valuable tool in examining the relationship between multiple substances, in conjunction with their cellular functions and in the context of various conditions such as T2D and response to the diet intervention. Preferably, a biomarker should be non-invasively accessible, low in cost, highly specific, sensitive and easy to interpret.

### Reporting

Study findings will be reported according to the Consolidated Standards of Reporting Trials guidelines. All findings, including null results, will be reported.

### Ethics and dissemination

#### Safety considerations

No harm directly related to the procedures or performance of the study is expected. However, minor discomfort, bleeding or bruising associated with self-administered blood sampling, and deterioration in laboratory or other examination values due to underlying disease, may occur. If an unforeseeable event occurs, participants are covered by patient insurance.

Any adverse events that are unresolved at the end of the study will be followed up by the investigator or the participant’s regular healthcare physician for as long as medically indicated, but without further recording in the study database.

A regulatory authority or the Swedish Ethical Review Authority may audit or inspect the study site, including source data verification. The purpose of such audits or inspections is to systematically and independently examine all study-related activities and documents to determine whether these activities were conducted, and data were recorded, analysed and accurately reported according to the protocol, good clinical practice and applicable regulatory requirements.

If test results indicate a previously undiagnosed disease or deterioration of a known disease, the information will be communicated to the participant via telephone or postal mail, as considered most appropriate. The participant will then be referred to their primary care physician for further management.

#### Ethical considerations

The study will be performed in accordance with ethical principles that have their origin in the Declaration of Helsinki and applicable regulatory requirements. The Swedish Ethical Review Authority has approved the study protocol, including the informed consent form, all advertising and any other written information and/or materials to be provided to participants, before the start of the study.

The study adheres to the Declaration of Helsinki and General Data Protection Regulation and has been approved by the Swedish Ethical Review Authority, including the main application, 2023-06622-01 (approved 24 January 2024), and applications regarding amendments, 2024-06668-02 (approved 4 November 2024), 2024-07357-02 (approved 1 December 2024) and 2025-01095-02 (approved 28 February 2025).

Substantial protocol changes will be documented in an amendment or revised protocol, registered at ClinicalTrials.gov, and approved by the Swedish Ethical Review Authority before implementation.

During the trial, personal identification data will be stored at *minforskning.se*. During the trial, personal identification data will be stored at *minforskning.se*, and data collected via questionnaires, dried blood spots and the *FoodSwitch* app will all be pseudonymised. After trial completion, the identification key will be stored securely and separately from study data to maintain pseudonymisation at all times.

#### Dissemination plan

Results will be published in peer-reviewed international scientific journals. Results of direct public interest may also be communicated to lay media in Sweden and/or abroad, directly or via press release.

The full study protocol, statistical code and deidentified individual participant data will be made available to qualified researchers on reasonable request. Requests will be considered on an individual basis and require a formal application outlining the research question, analysis plan and intended use. Data will be shared through a secure data transfer mechanism, following review and approval by the investigators.

## References

[1] Ong KL , Stafford LK , McLaughlin SA , et al . Global, regional, and national burden of diabetes from 1990 to 2021, with projections of prevalence to 2050: a systematic analysis for the Global Burden of Disease Study 2021. Lancet 2023;402:203–34. 10.1016/S0140-6736(23)01301-6 37356446 PMC10364581

[2] Eeg-Olofsson K , Åkesson K , Thorén A , et al . Nationella Diabetesregistret, årsrapport 2024 års resultat [Swedish National Diabetes Register, Year report 2024]. 2024.

[3] ElSayed NA , McCoy RG , Aleppo G , et al . 8. Obesity and Weight Management for the Prevention and Treatment of Type 2 Diabetes: Standards of Care in Diabetes–2025. Diabetes Care 2025;48:S167–80. 10.2337/dc25-S008 39651976 PMC11635032

[4] Ng M , Fleming T , Robinson M , et al . Global, regional, and national prevalence of overweight and obesity in children and adults during 1980–2013: a systematic analysis for the Global Burden of Disease Study 2013. Lancet 2014;384:766–81. 10.1016/S0140-6736(14)60460-8 24880830 PMC4624264

[5] Tang Y , Yang D . Overcoming dietary complexity in type 2 diabetes: influencing factors and coping strategies. Eur J Med Res 2025;30:82. 10.1186/s40001-025-02318-8 39910637 PMC11800452

[6] World Health Organization . Guiding principles and framework manual for front-of-pack labelling for promoting healthy diets. 2019.

[7] Siopis G , Wang L , Colagiuri S , et al . Cost effectiveness of dietitian-led nutrition therapy for people with type 2 diabetes mellitus: a scoping review. J Hum Nutr Diet 2021;34:81–93. 10.1111/jhn.12821 33280180

[8] Adolfsson ET , Rosenblad A , Wikblad K . The Swedish National Survey of the Quality and Organization of Diabetes Care in Primary Healthcare--Swed-QOP. Prim Care Diabetes 2010;4:91–7. 10.1016/j.pcd.2010.03.002 20434973

[9] Croker H , Packer J , Russell SJ , et al . Front of pack nutritional labelling schemes: a systematic review and meta-analysis of recent evidence relating to objectively measured consumption and purchasing. J Hum Nutr Diet 2020;33:518–37. 10.1111/jhn.12758 32364292

[10] Free C , Phillips G , Galli L , et al . The effectiveness of mobile-health technology-based health behaviour change or disease management interventions for health care consumers: a systematic review. PLoS Med 2013;10:e1001362. 10.1371/journal.pmed.1001362 23349621 PMC3548655

[11] Shariful Islam SM , Chow CK , Redfern J , et al . Effect of text messaging on depression in patients with coronary heart disease: a substudy analysis from the TEXT ME randomised controlled trial. BMJ Open 2019;9:e022637. 10.1136/bmjopen-2018-022637 PMC6398727 30787075

[12] The Swedish Internet Foundation . Svenskarna och internet 2019 [Swedes and the internet 2019]. 2019.

[13] McCarroll R , Eyles H , Ni Mhurchu C . Effectiveness of mobile health (mHealth) interventions for promoting healthy eating in adults: A systematic review. Prev Med 2017;105:156–68. 10.1016/j.ypmed.2017.08.022 28882743

[14] Coughlin SS , Whitehead M , Sheats JQ , et al . Smartphone Applications for Promoting Healthy Diet and Nutrition: A Literature Review. Jacobs J Food Nutr 2015;2:021. 26819969 PMC4725321

[15] Bretschneider MP , Kolasińska AB , Šomvárska L , et al . Evaluation of the Impact of Mobile Health App Vitadio in Patients With Type 2 Diabetes: Randomized Controlled Trial. J Med Internet Res 2025;27:e68648. 10.2196/68648 40344662 PMC12102620

[16] Storgärds M , Sundström J . New electronic informed consent including super safe signing and verification. Europe Biobank Week; 2020.

[17] Delfi Dabas . The product-/articledatabase for the swedish foodservice sector. n.d. Available: https://www.dabas.com/

[18] DLF and Delfi . Dagligvarukartan 2024, Sweden’s grocery retail market 2024. 2024.

[19] Kostråd till nyupptäckt: diabeteshandboken.se. 2024.

[20] Holmberg MJ , Andersen LW . Adjustment for Baseline Characteristics in Randomized Clinical Trials. JAMA 2022;328:2155–6. 10.1001/jama.2022.21506 36394881

[21] Wheelock ÅM , Wheelock CE . Trials and tribulations of ’omics data analysis: assessing quality of SIMCA-based multivariate models using examples from pulmonary medicine. Mol Biosyst 2013;9:2589–96. 10.1039/c3mb70194h 23999822

[22] Gustafson Hedov E , Nyberg F , Gustafsson S , et al . Person-Centered Web-Based Mobile Health System (Symptoms) for Reporting Symptoms in COVID-19 Vaccinated Individuals: Observational Study of System, Users, and Symptoms. JMIR Form Res 2024;8:e57514. 10.2196/57514 39476854 PMC11561448

[23] El Miedany Y , El Gaafary M , Youssef S , et al . Toward Electronic Health Recording: Evaluation of Electronic Patient-reported Outcome Measures System for Remote Monitoring of Early Rheumatoid Arthritis. J Rheumatol 2016;43:2106–12. 10.3899/jrheum.151421 27633823

[24] Shah KN , Hofmann MR , Schwarzkopf R , et al . Patient-Reported Outcome Measures: How Do Digital Tablets Stack Up to Paper Forms? A Randomized, Controlled Study. Am J Orthop (Belle Mead NJ) 2016;45:E451–7. 28005113

[25] Matthews M , Rathleff MS , Vicenzino B , et al . Capturing patient-reported area of knee pain: a concurrent validity study using digital technology in patients with patellofemoral pain. PeerJ 2018;6:e4406. 10.7717/peerj.4406 29568700 PMC5845563

[26] Boudreau SA , Badsberg S , Christensen SW , et al . Digital Pain Drawings: Assessing Touch-Screen Technology and 3D Body Schemas. Clin J Pain 2016;32:139–45. 10.1097/AJP.0000000000000230 25756558

[27] Ostler MW , Porter JH , Buxton OM . Dried blood spot collection of health biomarkers to maximize participation in population studies. J Vis Exp 2014;2014:50973. 10.3791/50973 PMC4091281 24513728

[28] Martinez P , Zemore SE . Feasibility of a mail-in, self-administered dried blood spot collection method in national, population-based alcohol surveys in the United States. Addiction 2019;114:1303–8. 10.1111/add.14603 30889308 PMC6548634

[29] Sakhi AK , Bastani NE , Ellingjord-Dale M , et al . Feasibility of self-sampled dried blood spot and saliva samples sent by mail in a population-based study. BMC Cancer 2015;15:265. 10.1186/s12885-015-1275-0 25886002 PMC4428002

[30] Quraishi R , Lakshmy R , Prabhakaran D , et al . Use of filter paper stored dried blood for measurement of triglycerides. Lipids Health Dis 2006;5:20. 10.1186/1476-511X-5-20 16839425 PMC1540415

[31] Affan ET , Praveen D , Chow CK , et al . Comparability of HbA1c and lipids measured with dried blood spot versus venous samples: a systematic review and meta-analysis. BMC Clin Pathol 2014;14:21. 10.1186/1472-6890-14-21 25045323 PMC4101836

[32] Hall JM , Fowler CF , Barrett F . blood collection devices: comparison of home prepared dried blood spots with standard venous blood analysis. Diabet Med 2020;37:1463–70. 10.1111/dme.14110 31418916 PMC7496699

[33] Scherf-Clavel M , Albert E , Zieher S , et al . Dried blood spot testing for estimation of renal function and analysis of metformin and sitagliptin concentrations in diabetic patients: a cross-sectional study. Eur J Clin Pharmacol 2019;75:809–16. 10.1007/s00228-019-02637-w 30706085

[34] Hebib L , Gustafson Hedov E , Storgärds M , et al . Digital tools and self-administered home blood tests: A convergent mixed methods pilot study. Digit Health 2025;11:20552076251365063. 10.1177/20552076251365063 40755958 PMC12317230

[35] Rawshani A , Rawshani A , Franzén S , et al . Risk Factors, Mortality, and Cardiovascular Outcomes in Patients with Type 2 Diabetes. N Engl J Med 2018;379:633–44. 10.1056/NEJMoa1800256 30110583

[36] Tsapas A , Avgerinos I , Karagiannis T , et al . Comparative Effectiveness of Glucose-Lowering Drugs for Type 2 Diabetes: A Systematic Review and Network Meta-analysis. Ann Intern Med 2020;173:278–86. 10.7326/M20-0864 32598218

[37] Mastronardi CA , Whittle B , Tunningley R , et al . The use of dried blood spot sampling for the measurement of HbA1c: a cross-sectional study. BMC Clin Pathol 2015;15:13. 10.1186/s12907-015-0013-5 26157353 PMC4495815

[38] Babu M , Snyder M . Multi-Omics Profiling for Health. Mol Cell Proteomics 2023;22:100561. 10.1016/j.mcpro.2023.100561 37119971 PMC10220275

[39] Al-Amrani S , Al-Jabri Z , Al-Zaabi A , et al . Proteomics: Concepts and applications in human medicine. World J Biol Chem 2021;12:57–69. 10.4331/wjbc.v12.i5.57 34630910 PMC8473418

[40] Guasch-Ferré M , Bhupathiraju SN , Hu FB . Use of Metabolomics in Improving Assessment of Dietary Intake. Clin Chem 2018;64:82–98. 10.1373/clinchem.2017.272344 29038146 PMC5975233

[41] Clish CB . Metabolomics: an emerging but powerful tool for precision medicine. Cold Spring Harb Mol Case Stud 2015;1:a000588. 10.1101/mcs.a000588 27148576 PMC4850886

