# Supplementary material for: Effect of the FoodSwitch application on type 2 diabetes in Sweden: a study protocol for the randomised controlled DIgitAl diabeTES Treatment – the Healthy Eating, heaLthy Patients trial (DIATEST-HELP)
Source: BMJ Open. 2025 Nov 16;15(11):e110141. doi: 10.1136/bmjopen-2025-110141 (PMC12917363; doi:10.1136/bmjopen-2025-110141)
Supplement: Supplementary data [file bmjopen-15-11-s001.pdf]

Research Participant Information

You are hereby asked to participate in a research study evaluating the effect of the FoodSwitch mobile application on long-term blood glucose (HbA1c) in type 2 diabetes. Participation involves finger-prick self-sampling with dried blood spots sent to researchers, completing questionnaires, and reporting symptoms via a digital application. The study is called "DIATEST".

Purpose

The study aims to investigate the effect of the FoodSwitch mobile application on long-term blood glucose (HbA1c) and other factors relevant to long-term complications of type 2 diabetes (blood lipids, renal function, low-grade inflammation). Symptoms and quality of life will also be evaluated.

Study Procedure

Upon digital consent via BankID/Freja eID through minforskning.se, you will access Symptoms, a digital system for self-reporting symptoms, function, and quality of life. You will complete questionnaires at baseline and after 6, 12, 18, and 26 weeks. These brief questionnaires cover health status, medication, lifestyle, and dietary habits. You may also map your symptoms on a three-dimensional figure at your own pace and frequency throughout the study. You will be asked whether you wish to share digital receipts from grocery purchases if available.

You will receive a lancet and filter paper by mail to your registered address with instructions for finger-prick self-sampling, applying blood drops to designated areas on the filter paper, allowing them to dry, and returning the filter papers in a prepaid envelope to researchers. This is performed at baseline and after 26 weeks.

Within two weeks of consent, you will complete digital questionnaires in Symptoms, share or send photos of grocery receipts on three occasions, measure waist circumference with a provided tape measure, and return the filter paper with self-collected blood samples.

After analysis, your HbA1c result will be provided on your personal page at minforskning.se. You will receive a second self-sampling kit and be asked to measure waist circumference at 26 weeks.

The FoodSwitch mobile application provides nutritional information on pre-packaged foods and suggests healthier alternatives. Products can be scanned in stores using the application. Half of participants will be randomized to use this "digital dietitian in your pocket" while the other half will receive standard primary care advice. Half of participants (regardless of FoodSwitch use) will also be randomized to receive a named contact person or access via a function mailbox.

If you participate, a copy of your data from minforskning.se and Symptoms will be transferred to researchers. Your data will be coded (personal identity number replaced with a

number) when transferred to the FoodSwitch technical platform if randomized to use the app. All results will be presented at group level and cannot be traced to you.

Risks

Processing personal data always involves privacy risks. These are minimized through two-factor authentication, encrypted databases and transfers, and modern secure communication standards. Access to the coded research database is restricted to few study personnel. Your data will be processed so that unauthorized persons cannot access it, in accordance with the General Data Protection Regulation (GDPR, 2016/679). Finger-prick sampling may cause mild discomfort.

Data Management

You may access and download all your data from the Symptoms system at any time. During the study, your data will be stored in an electronic database managed by the research group. Database access is restricted to few study personnel. Your personal identity number is stored separately from your data and will only be used by few research group members on rare occasions. Your data will be processed so that unauthorized persons cannot access it, in accordance with GDPR (2016/679). The data controllers are Linköping University and Uppsala University jointly. You have the right to free access to your data, correction of errors, deletion of identifiable data, and restriction of data processing. However, the right to deletion and restriction does not apply when data are necessary for the research. To access your data, contact dataskyddsombud@liu.se. If dissatisfied with data processing, you may file a complaint with the Swedish Authority for Privacy Protection (Datainspektionen).

Results

Research results will be presented in scientific journals and conferences and disseminated to the public via press releases. You will receive your HbA1c result via minforskning.se.

Voluntary Participation

Participation is voluntary and you may withdraw consent at any time via minforskning.se. You may continue using Symptoms regardless of study participation and may discontinue use at any time. If you choose not to participate or withdraw, you need not provide a reason and it will not affect your continued care or treatment.

Contact Information

Principal Investigator:
Karin Rådholm, Linköping University, Department of Health, Medicine and Caring Sciences (HMV), Division of Prevention, Rehabilitation and Community Medicine.
Phone: 0700-896651
Email: karin.radholm@liu.se

Consent to Participate

I consent to participate in the "DIATEST" study. I consent to the processing of my data as described above. I understand that participation is voluntary and I may withdraw at any time without explanation and without affecting my future care.

Ethics Review Authority Reference: 2023-06622-01
Date: 2023-10-28
