# Supplementary material for: Effect of the FoodSwitch application on type 2 diabetes in Sweden: a study protocol for the randomised controlled DIgitAl diabeTES Treatment – the Healthy Eating, heaLthy Patients trial (DIATEST-HELP)
Source: BMJ Open. 2025 Nov 16;15(11):e110141. doi: 10.1136/bmjopen-2025-110141 (PMC12917363; doi:10.1136/bmjopen-2025-110141)
Supplement: Supplementary data [file bmjopen-15-11-s002.pdf]

BMJ Publishing Group Limited (BMJ) disclaims all liability and responsibility arising from any reliance placed on this supplemental material which has been supplied by the author(s)

Forskningspersonsinformation

Du tillfrågas härmed om deltagande i en forskningsstudie som handlar om att utvärdera vilken effekt mobilapplikationen *FoodSwitch* har på långtidsblodsocker (HbA1c-värde) vid typ 2 diabetes. Deltagande i studien innebär att du ska sticka dig själv i fingret och posta torkade bloddroppar till forskarna och att du ska svara på frågeformulär och ange eventuella besvär i en digital applikation. Studien heter ”DIATEST”.

Syfte

Syftet med studien är att undersöka vilken effekt mobilapplikationen *FoodSwitch* har på långtidsblodsocker (HbA1c-värde) och andra viktiga faktorer som kan ha inverkan när det gäller långtidskomplikationer (såsom blodfetter, njurfunktion och låggradig inflammation) av typ 2 diabetes. Vi vill även utvärdera symptom och livskvalitet.

Etikprövningsmyndigheten
2023-06622-01-473081
2023-10-28

Hur går studien till?

Om du samtycker till deltagande, vilket sker digitalt med hjälp av BankID/Freja eID via portalen minforskning.se, kommer du att få tillgång till ett digitalt system för självrapportering av symtom, funktion och livskvalitet, *Symptoms.* I det digitala systemet ombeds du besvara frågeformulär vid studiestart, samt efter 6, 12, 18 respektive 26 veckor. Enkäter är korta frågeformulär om hälsotillstånd, medicinering, levnadsvanor och kostvanor. Utöver detta får du i egen takt och utifrån egna önskemål kartlägga dina symtom och rita dem på en tredimensionell figur i systemet. Om du vill kan du fortsätta kartlägga förändringar i ditt mående upprepat över tid , så ofta som du önskar, under den tid som studien pågår. Du

af Geijerstam P, *et al. BMJ Open* 2025; 15:e110141. doi: 10.1136/bmjopen-2025-110141

kommer att bli tillfrågad om du vill dela digitala kvitton från matinköp om du har tillgång till sådan data.

Du kommer också att få en lansett och filterpapper hemskickade i ett kuvert till din folkbokföringsadress med instruktioner om hur du ska sticka dig själv i ett finger (eller be en närstående om hjälp med det) och droppa några droppar blod på angivna ställen på filterpapperet, låta detta bloddropparna torka och sedan skicka filterpapperen i ett för-frankerat returkuvert till forskarna. Detta görs vid studiestart och efter 26 veckor (ett halvår).

Etikprövningsmyndigheten
2023-06622-01-473081
2023-10-28

Inom två veckor efter att du samtyckt till att delta i studien ombeds du besvara digitala enkäter i *Symptoms*, dela alternativt skicka foton av eventuella kvitton för matinköp vid tre tillfällen, mäta midjeomfång med ett måttband som skickas till dig och returnera filterpapper med självadministrerat blodprov.

När forskarna har analyserat bloddropparna och sett att du har fyllt i frågeformuläret i *Symptoms* kommer du att få svar på blodprovet av långtidsblodsocker (HbA1c) på din personliga sida på studiens websida, (minforskning.se).

Du kommer att få ett paket med ett andra självprovtagningspaket för blodprov och medföljande instruktioner och ombes att mäta midjeomfång vid 26 veckor efter studiestart.

Etikprövningsmyndigheten
2023-06622-01-473081
2023-10-28

Mobilapplikationen *FoodSwitch* ger information om näringsinnehåll i färdigförpackade livsmedel och föreslår hälsosammare alternativ. Förpackade produkter kan enkelt skannas i mataffären med hjälp av applikationen. Hälften av deltagarna kommer att slumpas till att använda denna ”digitala dietist i fickan” och andra hälften kommer att få standardråd som ges i primärvården. Hälften av deltagarna (oberoende om de använder FoodSwitch eller inte) kommer också att slumpas till att få en namngiven kontaktperson för frågor eller att få möjlighet att ta kontakt via en funktionsbrevlåda.

Om du vill medverka i detta forskningsprojekt kommer en kopia av dina data i minforskning.se och *Symptoms* att överföras till forskarna. Data om dig är kodade (dvs ditt personnummer är utbytt mot en siffra som inte identifierar dig) när de överförs till FoodSwitch tekniska plattform om du slumpas till att använda appen FoodSwitch. Alla resultat kommer att presenteras på gruppnivå och de kommer inte att kunna härledas till dig.

Vilka risker finns?

Att hantera personuppgifter medför alltid en integritetsrisk. Denna risk minimeras genom de skyddsåtgärder som används i projektet, såsom tvåfaktorsautentisering för inloggning, krypterade databaser och överföringar samt moderna standarder för säker kommunikation. Åtkomsten till den kodade forskningsdatabasen är begränsad till ett fåtal personer som ansvarar för studien. Dina uppgifter kommer att behandlas så att obehöriga inte kan ta del av dem, i enlighet med Dataskyddsförordningen (GDPR, 2016/679). Att sticka sig i fingret med en lansett och hantera en bloddroppe kan medföra lätt obehag.

Vad händer med mina uppgifter?

Du kan när som helst ta del av och ladda ner alla dina data ur *Symptoms*-systemet. Under studien kommer uppgifter om dig att lagras i en elektronisk databas som hanteras av forskargruppen. Åtkomsten till databasen är begränsad till ett fåtal personer som ansvarar för studien. Ditt personnummer lagras separat från dina data och kommer bara att användas av ett fåtal personer i forskargruppen vid enstaka tillfällen. Dina uppgifter kommer att behandlas så att obehöriga inte kan ta del av dem, i enlighet med Dataskyddsförordningen (GDPR, 2016/679). Ansvarig för dina personuppgifter i studien är Linköpings universitet och Uppsala universitet gemensamt. Du har rätt att kostnadsfritt få ta del av de uppgifter om dig som hanteras i studien och få eventuella fel rättade vid behov. Du kan också begära att uppgifter som kan kopplas till din identitet raderas samt att behandlingen av dina personuppgifter begränsas. Rätten till radering och till begränsning av behandling av personuppgifter gäller dock inte när uppgifterna är nödvändiga för den aktuella forskningen. Om du vill ta del av uppgifterna kan du kontakta dataskyddsombud@liu.se. Om du är missnöjd med hur dina personuppgifter behandlas har du rätt att ge in klagomål till Datainspektionen, som är tillsynsmyndighet.

Etikprövningsmyndigheten
2023-06622-01-473081
2023-10-28

Resultat

Forskningsresultaten kommer att presenteras i vetenskaplig tidskrift och vid forskningskonferens och kommer att delges allmänheten via pressmeddelanden. Du kommer att få ta del av ditt HbA1c-provsvar via portalen minforskning.se.

Frivillighet

Ditt deltagande i studien är frivilligt och du kan när som helst välja att avbryta deltagandet

genom att dra tillbaka ditt samtycke på minforskning.se. Du kan fortsätta använda systemet *Symptoms* oavsett om du är med i forskningsstudien eller inte och du kan när som helst avsluta din användning av det systemet. Om Du väljer att inte delta eller om du vill avbryta ditt deltagande behöver du inte uppge varför och det kommer inte heller att påverka din fortsatta vård eller behandling.

Kontaktuppgifter till ansvarig forskare

Karin Rådholm, Linköpings universitet, Institutionen för hälsa, medicin och vård (HMV), Avdelningen för prevention, rehabilitering och nära vård.

Telefonnummer: 0700-896651

E-post: karin.radholm@liu.se

Etikprövningsmyndigheten
2023-06622-01-473081
2023-10-28

Samtycke till att delta i studien

Jag samtycker till att delta i studien “DIATEST”. Jag samtycker till att uppgifter om mig behandlas på det sätt som beskrivs i informationen ovan. Jag känner till att mitt deltagande är helt frivilligt och att jag när som helst och utan närmare förklaring kan avbryta mitt deltagande utan att detta påverkar mitt framtida omhändertagande.

af Geijerstam P, *et al. BMJ Open* 2025; 15:e110141. doi: 10.1136/bmjopen-2025-110141
